# A Systematic Literature Review of the Association of Lipoprotein(a) and Autoimmune Diseases and Atherosclerosis

**DOI:** 10.1155/2012/480784

**Published:** 2012-12-05

**Authors:** I. Missala, U. Kassner, E. Steinhagen-Thiessen

**Affiliations:** Department of Lipid Disorder, Charité University Medical Department in Berlin, Berlin, Germany

## Abstract

*Objective*. To investigate the association of lipoprotein(a) and atherosclerosis-related autoimmune diseases, to provide information on possible pathophysiologic mechanisms, and to give recommendations for Lp(a) determination and therapeutic options. *Methods*. We performed a systematic review of English language citations referring to the keywords “Lp(a)” AND “autoimmune disease” AND “atherosclerosis,” “Lp(a)” AND “immune system” OR “antiphospholipid (Hughes) syndrome (APS)” OR “rheumatoid arthritis” OR “Sjögren's syndrome” OR “systemic lupus erythematosus” OR “systemic sclerosis” OR “systemic vasculitis” published between 1991 and 2011 using Medline database. *Results*. 22 out of 65 found articles were identified as relevant. Lp(a) association was highest in rheumatoid arthritis (RA), followed by systemic lupus erythematosus (SLE), moderate in APS and lowest in systemic sclerosis (SSc). There was no association found between Lp(a) and systemic vasculitis or Sjögren's syndrome. *Conclusion*. Immune reactions are highly relevant in the pathophysiology of atherosclerosis, and patients with specific autoimmune diseases are at high risk for CVD. Elevated Lp(a) is an important risk factor for premature atherosclerosis and high Lp(a) levels are also associated with autoimmune diseases. Anti-Lp(a)-antibodies might be a possible explanation. Therapeutic approaches thus far include niacin, Lp(a)-apheresis, farnesoid x-receptor-agonists, and CETP-inhibitors being currently under investigation.

## 1. Introduction

Atherosclerosis is a major cause of cardiovascular disease (CVD). Autoimmune reactions and inflammation are mainly involved in their pathogenesis. Already at early onset atherosclerosis inflammatory cells (monocytes, macrophages, dendritic cells, T- and B-cells) and cytokines can be identified in the lesion area and those cells may provoke cell-mediated immune reactions (CMIR) that (i) modulate the development of atherosclerosis and may (ii) predetermine its progression [1, 2]. Immune reactions may modulate atherosclerosis in different ways: (i) *β*2 glycoprotein I-immunization led to an increase, (ii) heat shock protein (HSPs) 60/65 antigen led to an increase, and (iii) oxLDL-immunization led to a decrease [3, 4]. In addition to established risk factors of CVD, autoimmune processes are discussed as being highly relevant. Autoimmune disorders are associated with a high CVD risk in clinical practice. In a major autoimmune disease, SLE, animal studies identified mainly proinflammatory Th1 cytokines (e.g., IFN-gamma), whereas in humans with SLE mainly Th2 cytokines were identified as involved in CMIR [3]. Risk factors for CVD in SLE are enhanced atherosclerosis, increased inflammation, elevated levels of oxidized LDL (oxLDL) and autoantibodies against oxLDL, increased triglycerides, total cholesterol (TC) and Lp(a) and decreased HDL-cholesterol, raised systemic inflammation and the presence of anti-phospholipid antibodies (aPL), high homocysteine levels, and osteoporosis [2]. But the relative risk of CVD differs among the specific autoimmune disease. Some autoimmune disorders like systemic lupus erythematosus (SLE), rheumatoid arthritis, antiphospholipid (Hughes) syndrome (APS), and systemic sclerosis carry a high risk of CVD development, whereas others as the Sjögren's syndrome and systemic vasculitis seem to have a weaker influence on CVD development.

Lp(a) remains an important risk factor for premature atherosclerosis and CVD development. It was first mentioned by Berg, K 1963 [5] and contains an LDL-like particle with apolipoprotein B-100 (apoB 100) linked to apolipoprotein(a) (apo(a)). Lp(a) distribution in population is lowest in Caucasians, modest in Hispanics, Chinese, Japanese, and highest in Blacks [6]. Multiple existing genetic variations and polymorphisms of apo(a) cause variations in population Lp(a) plasma levels. Lp(a) is involved in atherosclerosis in different ways: (i) it accumulates in the arterial intima and (ii) it activates inflammatory cells and (iii) binds to proinflammatory-oxidized-phospholipids [7, 8]. It additionally promotes thrombosis and inhibits fibrinolysis due to the high structural homology between apo(a) and plasminogen [8, 9]. It is secreted by the liver and undergoes renal and hepatic metabolism.

There is evidence that serum Lp(a) and LDL can act additively in the development of coronary heart disease. Lp(a) undergoes oxidative modification like oxLDL [10] and provokes an immune response [11]. It enters the arterial wall via a macrophage scavenger receptor [12]—a known pathway for ox-LDL. Ox-LDL-formation induces the production of anti-ox-LDL-antibodies, and a similar mechanism is suggested for ox-Lp(a) [11, 13]. These autoantibodies have been found in atherosclerotic lesions [14]. Furthermore, antibodies against ox-LDL and ox-Lp(a) are more prevalent in patients with specific autoimmune diseases [11, 15, 16].

Lp(a) is involved in immunological processes and several studies showed a high association with some autoimmune diseases. Mechanisms mainly involved in this association are HLA-genotype-predominance, Lp(a)-autoantibodies, the relation of fibrinolytic system parameters and Lp(a), the relation of acute phase system parameters and Lp(a), and the complex formation of beta(2)-GPI-Lp(a).

Our aim was to show that patients with specific autoimmune disorders have a higher atherosclerosis risk which might be aggravated by elevated Lp(a) levels. The measurement of Lp(a) levels might be an additional tool to identify patients at high risk for CVD.

## 2. Methods 

### 2.1. Search Strategy

A systematic literature research was conducted consulting the Medline database PUBMED using the following keywords: “Lp(a)” AND “autoimmune disease” AND “atherosclerosis,” “Lp(a)” AND “immune system,” “Lp(a)” AND “antiphospholipid (Hughes) syndrome (APS,)” “Lp(a)” AND “rheumatoid arthritis,” “Lp(a)” AND “Sjogren's syndrome,” “Lp(a)” AND “systemic lupus erythematosus,” “Lp(a)” AND “systemic sclerosis,” “Lp(a)” AND “systemic vasculitis.”

### 2.2. Selection Criteria

Titles and abstracts were excluded (Table 1) if they were (i) unrelated to topical Lp(a) and autoimmune disease and atherosclerosis or CVD, (ii) not written in English, (iii) unpublished studies, (iv) only available as abstracts and not as full-text articles (reprints were requested), (v) theses or book chapters, (vi) investigations published in nonpeer-reviewed journals, (vii) single case studies, and (viii) highly specific articles not considered as relevant in this context.

### 2.3. Manual Review

All full-text articles were read by two independent reviewers and rated as “relevant” or “not relevant” in this context. Additionally a search of secondary sources as articles references was committed.

## 3. Results

**Figure 1:**
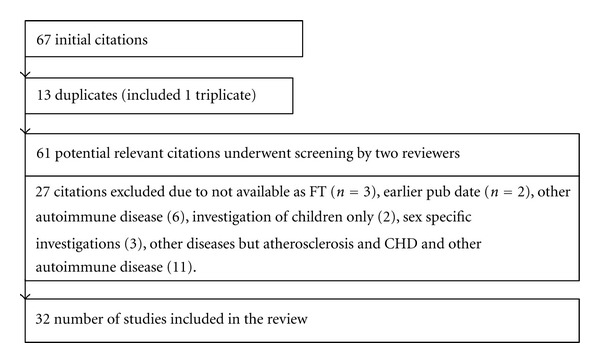
Systematic review process.

The initial search yielded 67 citations, there were 25 articles for “Lp(a)” AND “rheumatoid arthritis,” 20 for “systemic lupus erythematosus,” 4 for “immune system,” 11 for “APS,” 3 for “systemic sclerosis”, and 4 for “autoimmune disease.” The literature research showed no citations for the keywords “systemic vasculitis” and “Sjögren's syndrome.” Thirteen citations (Frostegard, Sari, Romero 2000 (3), Zhang 2011, Atsumi, Sakata) were duplicates. From the remaining 61 citations 32 met our inclusion criteria and were identified as relevant. 27 articles were excluded, because of earlier publication date (2), no full text (FT) available (3), other autoimmune diseases (6), investigation of children only (2), sex specific investigations (3), other diseases but atherosclerosis and CHD and other autoimmune diseases (11).  Figure 1 shows a flowchart of the systematic review process.

### 3.1. Lp(a) and Systemic Sclerosis (SSc)

SSc is an autoimmune disorder characterized by excessive production of collagen, fibronectin, and other matrix proteins which accumulate in the skin and internal organs with resulting thrombosis [17]. Abnormally high Lp(a) levels are found in SSc patients leading to defective fibrinolysis and a hypercoagulable state [18]—endothelial injury worsens the situation even more. Ames et al. [17] showed significantly elevated Lp(a) levels in SSc patients versus controls resulting in a hypercoagulable state with elevated plasma levels of fibrinogen and von Willebrandt Factor (VWF) due to (i) defective tissue plasminogen activator (tPA) release and (ii) increased tPA inhibitor concentrations and (iii) by increased thrombin generation with enhanced fibrin formation. A possible explanation is that apo(a) has high sequence homology with plasminogen. It might compete with tPA for fibrin binding and therefore weakens fibrinolysis [19].

Lippi et al. [20] showed statistically significant differences in SSc patients when compared to healthy controls in Lp(a) levels. They concluded that Lp(a) measurement might be useful in SSc to identify and eventually treat subsets of patients more predisposed to develop thrombotic complications.

### 3.2. Lp(a) and Rheumatoid Arthritis (RA)

RA is a chronic, systemic, inflammatory disorder that primarily involves joints. It is a polyarticular disease with a gradual onset, intermittent or migratory joint involvement, and a monoarticular onset are different types of clinical presentations of RA. In addition, extra-articular manifestations may be present.

Higher Lp(a) levels are found in RA patientswhen compared to healthy controls. Asanuma et al. [21] showed significantly higher Lp(a) values in RA patients compared to controls. Additionally they showed a high predominance of the S3 allele and concluded causality. Lee et al. [22] found elevated Lp(a) levels in RA patients compared to controls as well. Although Lp(a) tended to be higher in RA, they could not find a distinct acute phase pattern of Lp(a). Their data support the phenomenon that dyslipoproteinemia observed in RA is associated with inflammation. Dursuno et al. [23] showed a positive correlation between Lp(a) and CPR level and a negative correlation between Lp(a) and HDL level in RA patients. They concluded that infammationa processes in RA patients my cause both changes in Lp(a) and HDL-C metabolism. Wang et al. [24] reported that Lp(a) and ox-Lp(a) concentrations in active RA were higher than those in both inactive RA and control; Lp(a)-immune complex (IC) concentrations in active RA were also higher than inactive RA, while no difference was found in Lp(a), ox-Lp(a), and Lp(a)-IC concentrations between inactive RA and controls. Lp(a), ox-Lp(a), and Lp(a)-IC were all found positively related with elevated C-reactive protein (CRP) levels and erythrocyte sedimentation rate (ESR), respectively. The study results underline the role of Lp(a) as acute phase protein.

Kerekes et al. [25] discovered low flow mediated dilatation (FMD) and high Carotid intima-media-thickness (ccIMT) in RA patients and a correlation of Lp(a) in RA-associated atherosclerosis. They suggest the evaluation of FMD% and ccIMT as useful tool to assess RA patients with high cardiovascular risk.

Zhang et al. [26] showed a complex formation of beta(2)-glycoprotein I (beta(2)-GPI) with Lp(a) in patients with active RA. Inflammation and oxidative stress in RA contribute to the increase of ox-Lp(a) and subsequently the formation of beta(2)-GPI-Lp(a). Beta(2)-GPI-Lp(a) and beta(2)-GPI-ox-LDL complex concentrations increased in RA patients and may be useful in assessing the development of atherosclerosis in patients with autoimmune diseases. Inflammation and oxidative stress may result in increased ox-Lp(a) and ox-LDL, and subsequently the formation of the complexes of beta(2)-GPI-Lp(a) and beta(2)-GPI-ox-LDL.

### 3.3. Lp(a) and Systemic Lupus Erythematosus (SLE)

SLE is a classic autoimmune disease characterized by the production of autoreactive T cells and autoantibodies that may affect multiple organ systems. SLE patients were found to have elevated serum Lp(a) levels compared to healthy controls in several studies [15–17], and developed preferably myocardial infarction [18]. Premature atherosclerosis and coronary artery disease (CAD) in SLE-disorder have been reported as major cause of mortality.

The risk of CVD development in SLE-patients is very high, SLE-related CVD is a common phenomenon. The risk factors for CVD in SLE include [2] atherosclerosis (ccIMT), raised oxLDL and autoantibodies to oxLDL, combined dyslipidemia with high triglyceride and low HDL, Lp(a), raised systemic inflammation, presence of anti-phospholipid antibodies including lupus anticoagulant, homocysteine-levels, and more frequent osteoporosis. Sari et al. [3] showed in their investigation that SLE patients have a risk of developing coronary artery disease which is associated with high levels of serum TC and Lp(a) and low levels of HDL-C and apo A-I.

McMahon et al. [27] showed that high leptin levels greatly increase the risk of subclinical atherosclerosis in SLE and are also associated with an increase in inflammatory biomarkers of atherosclerosis such as HDL, Lp(a), and oxPL/apoB100. High leptin levels may help to identify patients with SLE at risk of atherosclerosis. Zhang et al. [28] reported about the existence of beta(2)-GPI-Lp(a) complexes in both controls and SLE patients. The complexes levels are increased in SLE patients.

 There was high association between elevated Lp(a) and lupus erythematosus with renal involvement [29]—lupus nephritis was shown in 30 patients [30] compared to SLE-patients without renal failure. Lp(a) was increased in patients with proteinuria [31]. Several studies revealed a positive correlation between serum Lp(a) and serum cholesterol and urinary protein levels, and an inverse correlation between Lp(a) and albumin levels [3, 32, 33]. For patients with lupus anticoagulant besides the elevated Lp(a) a higher concentration of activated factor VII (FVIIa) was shown worsening the prothrombotic state of the disease [34]. Systemic lupus erythematosus patients had higher leptin levels, and there was a significant correlation between leptin level and Lp(a) [27].

### 3.4. Lp(a) and Antiphospholipid Syndrome (APS)

Antiphospholipid syndrome (APS) is an autoimmune disease characterized by arterial and/or venous thrombosis and recurrent abortions, accompanied by elevated titers of antiphospholipid antibodies [35]. Significantly higher plasma levels of Lp(a) are found in patients with APS [36–38]. Kritz et al. [39] demonstrated an association in patients with antiphospholipid syndrome as well as those with lack in the prostacyclin synthesis stimulating plasma factor (PF) and Lp(a), indicating a biochemical interaction.

Romero et al. [13] demonstrated the existence of autoantibodies against malondialdehyde (MDA)-Lp(a) in APS. The presence of antibodies reacting not only against MDA-LDL but also against MDA-Lp(a) supports the hypothesis of a role for oxidative phenomena in the pathogenesis of APS and atherosclerosis.

Yasuda et al. [40] focussed on the association of beta(2)-GPI and lipoprotein metabolism in APS patients, they showed individuals with heterozygous beta(2)-GPI deficiency showed significantly lower concentrations of serum beta(2)-GPI, but no significant influence on lipid metabolism was found.

López Lira [41] mentioned in their study that the interference in the plasmin conversion by anti-beta2GPI antibodies could generate thrombosis as observed in APS.

Bećarević et al. [38] investigated the appearance of recurrent cardio- and cerebrovascular events in patients with APS. They showed that only patients with stroke had a recurrence of cerebrovascular episodes (this was not shown for patients with myocardial infarction). Their conclusion was that measurement of apo(a) concentrations will help in the followup of those patients and thus in the prediction of future episodes.

Table 2 shows the design characteristics and Key messages of the included studies.

## 4. Discussion

This structured, systematic literature review identified 22 relevant studies related to the association of Lp(a) with specific atherosclerosis-related autoimmune diseases. Most of the investigations were randomized controlled trials with patients suffering from one specific autoimmune disease, there were just few reviews focusing on the association of atherosclerosis and immune system/autoimmune disorder in general.

Objectives of interest were HLA-genotype-predominance, Lp(a)-autoantibodies, relation of fibrinolysis system parameters and Lp(a), relation of acute phase system parameters and Lp(a), and complex formation of  beta(2)-GPI-Lp(a) and Lp(a)-apheresis. In general study results were concurrent in their overall message highlighting the occurrence of elevated Lp(a) levels in active autoimmune disease. All articles emphasized the influence of autoimmune mechanism on lipid metabolism esp. the occurrence of oxLDL and ox-Lp(a). Oxidation of LDL and Lp(a) is postulated to play a key role in the early initiation of atherosclerosis. Also changes in lipoproteins due to glycosylation, like the formation of beta(2)-GPI-Lp(a) [26] which were first detected in patients with RA and APS [28, 40, 41], and then in patients with CAD [42], might lead to early atherosclerosis. 

Apart from its proatherogenic potential Lp(a) has also thromboembolic properties due to the structural analogy of apo(a) and plasminogen. In SSc patients Lp(a) directly weakened the fibrinolytic process by competition with tPA for fibrin binding leading to clinical apparent increased risk and occurrence of thrombosis [17]. We assume that in clinical practice in SSc patients Lp(a) level should be measured to evaluate their thrombosis risk and initiate a sufficient preventive treatment.

Furthermore Lp(a) elevation merges with acute-phase-protein increase. In RA patients Lp(a) was associated with elevated CRP-level and erythrocyte sedimentation rate (ESR) and therefore playing an important role in the acute phase cascade reaction process [23] Lp(a) is claimed to react as acute phase protein in other diseases as well esp. ischemic stroke [43] vestibular neuronitis [44], in patients on chronic haemodialysis [45, 46] and polymyalgia rheumatica [47]. 

The impact of glycosylation in atherosclerosis development with complex formation of beta(2)-GPI with Lp(a) mentioned above was shown for RA patients. Wang et al. [24] investigated the association of Lp(a)-beta(2)-GPI-complexes and coronary artery disease; they showed that ox-Lp(a) was a risk factor only for acute coronary syndrome, while not for stable coronary artery disease. Beta(2)-GPI-Lp(a) levels were found to be positively associated with Lp(a), ox-Lp(a), maximal stenosis, and a number of vascular diseases in patients with ACS or stable CAD, respectively. They suggest that high levels of beta(2)-GPI-Lp(a) are associated with the presence and severity of CAD and may be a strong risk factor for atherosclerosis. An association in APS patients was shown as well [40]. This should be object of further research. 

The formation of autoantibodies towards Lp(a) seems to be triggered by autoimmune diseases. Anti-malondialdehyde (MDA)-Lp(a) were detected in patients with APS syndrome [13]. The fact that Lp(a) is recognized as “antigen” might be a reaction to the changes it undergoes due to oxidation and glycosylation. This phenomenon was detected in APS and SLE patients as well. 

Interestingly Lp(a)'s association with specific HLA-DR genotypes has been controversially discussed [1, 48]. Jonasson et al. [1] found no correlation between Lp(a) level and atherosclerosis and certain HLA genotypes in their investigation with 50 early onset atherosclerosis patients versus CAD patients. Dahlén [48] have shown an association for 30 patients between concurrent infectious disease and mean Lp(a) level. They suggest that an autoimmune process may especially occur in individuals with inherited high Lp(a) levels and certain HLA class II genotypes, triggered by a concurrent infection.

### 4.1. Treatment Approaches and Recommendations

#### 4.1.1. Niacin

Nicotinic acid (1–3.0 g/day) reduces Lp(a) levels up to 35–40% [49] and might be considered if, in addition to LDL-cholesterol lowering, decreases in triglycerides and Lp(a) and increases in HDL-cholesterol are aimed [26]. So far there is only one study investigating the effects of niacin as added on therapy versus atorvastatin alone on intima-media-thickness and Lp(a) level [50]. In 30 men with CHD and Lp(a) excess a regression of CIMT on an average of 0.06 mm in 6 months was shown. Additionally a reduction of Lp(a) from 84 ± 40  to  67 ± 25 mg/dL occurred after 6 weeks and up to 65 ± 37 mg/dL after 6 months of treatment, *P* < 0.01. This is a hint that niacin might be useful in patients with elevated Lp(a) and CHD.

Niacin treatment alone or in combination with other lipid lowering agents showed cardiovascular benefits in several studies [51–53].

#### 4.1.2. Lp(a)-Apheresis

Lp(a)-apheresis might be a promising therapeutic approach for patients with rare autoimmune diseases without treatment alternative, CVD progression and highly elevated Lp(a) levels [54, 55]. Jaeger et al. [56] showed in a longitudinal, multicenter, cohort-study with 120 patients a median reduction of Lp(a) concentration from 4.00 micromol/L to 1.07 micromol/L with apheresis treatment (*P* < 0.0001). Hovland et al. [57] investigated in a recently published prospective cross-over study with 3 FH-patients the effect of weekly lipid apheresis with three different columns: DL-75, LA-15 and EC-50W on Lp(a) levels. They showed an average reduction of Lp(a) by 70%, 74%, and 75% (all *P* < 0.0001) for DL-75, LA-15 and EC-50 W. Decision making of lipid apheresis should be based on CVD-progress, LDL cholesterol (LDL-C), or Lp(a) level if optimal conservative therapy is applied (lifestyle and maximal lipid-lowering drug therapy) [58].

#### 4.1.3. Other Agents

New promising approaches contain thyroid hormone analogues, Apo-B-synthesis inhibitors, Farnesoid X receptor Agonists [59, 60], and CETP inhibitors-being currently under investigation.

## 5. Conclusion

We have shown an association between specific autoimmune disorders and elevated Lp(a) levels and the development of atherosclerosis. Lp(a) increase in autoimmune disease might play an important role as prognosis worsening risk factor of atherosclerosis and CHD. 

Therefore it could be assumed that the Lp(a) measurement in patients with autoimmune disease is a worthwhile objective to investigate their atherosclerosis and CVD development risk.

## Figures and Tables

**Table 1:** Exclusion and inclusion criteria of review.

Exclusion	Inclusion
Unrelated to topical Lp(a) and autoimmune disease and atherosclerosis and CVD considered as not relevant	Autoimmune disorders: antiphospholipid (Hughes) syndrome (APS), rheumatoid arthritis, Sjögren's syndrome, systemic lupus erythematosus, systemic sclerosis, and systemic vasculitis
Unpublished studies	Published between 1991 and 2011
Single case studies	
Available not as full text (reprints were requested)	
Dissertations or theses or book chapters	

**Table 2:** Design characteristics and messages of included studies.

Objective	Reference	Results
APS	Kritz et al. 1996 [39]	All patients with a deficient PF activity had high Lp(a) values. While the prevalence of PF deficiency ranges about 1-2%, in 7 (19%) patients with clinically manifested atherosclerosis and 3 (19%) healthy adults with elevated Lp(a) this defect was found. The findings demonstrate an association between PF deficiency and Lp(a), indicating a biochemical interaction.

APS	Yasuda et al. 2000 [40]	A low concentration of beta(2)-GPI seemed not to be associated with apparent abnormality in lipoprotein metabolism.

APS	Bećarević et al. 2007 [38]	PAPS patients with cerebrovascular insults had recurrence of cerebrovascular episodes, measurement of apo(a) concentrations will help in the follow-up of those patients and thus in the prediction of future episodes.

APS SLE	Atsumi et al. 1998 [36]	Elevated plasma levels of Lp(a) were found in patients with APS compared to 22 healthy controls (*P* = 0.0001). Patients with APS with maximal elevation of Lp(a) showed a lower fibrinolytic activity (lower D-dimer and higher plasminogen activator inhibitor) than patients whose Lp(a) was within a normal range. These findings suggest that Lp(a) may represent a marker of APS and that Lp(a) has a negative effect on the fibrinolytic system that might contribute to the thrombotic tendency of APS.

APS	Romero eta al. 1999 [13]	Existence of autoantibodies against MDA-Lp(a). The presence of antibodies reacting not only against MDA-LDL but also against MDA-Lp(a) supports the hypothesis of a role for oxidative phenomena in the pathogenesis of APS and atherosclerosis.

APS SLE immune	Romero et al. 2000 [11]	A number of factors can be encountered in the pathogenesis of the accelerated arterial disease seen in patients with antiphospholipid (Hughes) syndrome (APS) and systemic lupus erythematosus (SLE). Among these, high levels of Lp(a) have been described in both and increasing evidence indicates that patients with antiphospholipid antibodies (aPL) are under oxidative stress. Recent studies suggest that the so-called “oxidation theory of atherosclerosis” may also be applied to Lp(a).

APS	López-Lira et al. 2006 [41]	Beta2-glycoprotein I (beta2GPI) is a glycoprotein of unknown physiological function. It is the main target antigen for antiphospholipid antibodies in patients with antiphospholipid syndrome (APS). beta2GPI binds with high affinity to the atherogenic lipoprotein Lp(a) which shares structural homology with plasminogen, a key molecule in the fibrinolytic system. Impaired fibrinolysis has been described in APS.

APS	Yamazaki et al. 1994 [37]	Patients with aPL are in hypercoagulable state. High levels of Lp(a) in plasma may impair the fibrinolytic system resulting in thromboses, especially in the arterial system.

RA	Asanuma et al. 1999 [21]	The mean serum Lp(a) level was significantly higher (*P* < 0.001) in the RA patients (27.5 mg/dL) than in the controls (15.0 mg/dL) possibly partly because of S3 phenotype predominance.

RA	Busso et al. 2001 [61]	These data indicated that synovial fluid apo(a) originates from circulating Lp(a) and that diffusion of Lp(a) through synovial tissue is facilitated in inflammatory types of arthritis. In synovial tissues, apo(a) co-localized with fibrin. In humans, apo(a) may modulate locally the fibrinolytic activity and may thus contribute to the persistence of intra-articular fibrin in inflammatory arthritis.

RA	Cesur et al. 2007 [62]	Analysis of the six studies showed Lp(a) level was higher and HDL level was lower in RA patients than in healthy controls. Patients with RA may have altered lipid profiles from one country to another one. Especially in Turkey, higher serum Lp(a), lower HDL-C and higher TG levels may be found in RA patients instead of some findings of other countries showing different results. Ethnicity may be a reason for these findings.

RA	Dahlén 1994 [48]	In this paper additional results of interleukin determinantions in relation to HLA type and Lp(a) levels are presented and discussed. It is suggested that an autoimmune process, perhaps triggered by a concomitant intracellular infection may occur, especially in patients with inherited high Lp(a) levels in combination with certain inherited HLA class II genotypes.

RA	Dahlén 1994 [48]	The associations found between LP(a) and insulin release, rheumatoid arthritis and renal diseases suggest that Lp(a) may be involved in immunological mechanisms. In a new hypothesis it is suggested that an autoimmune process might especially occur in individuals with inherited high Lp(a) levels and certain HLA class II genotypes, triggered by a concurrent infection.

RA	Wållberg-Jonsson et al. 1995 [47]	Certain HLA class II DR genotypes in combination with high Lp(a) levels and C. pneumoniae titers occurred more frequently in both male and female patients than in controls.

RA	Dursunoğlu et al. 2005 [23]	In the RA and control groups, serum Lp(a) levels were 39.2 ± 20.6 mg/dL and 14.8 ± 9.7 mg/dL, respectively (*P* < 0.001). CRP levels were positively correlated with Lp(a), HDL-C level were negatively correlated with Lp(a) (*r* = −0.36, *P* < 0.001).

RA	Zrour et al. 2011 [63]	Sera of patients showed higher TC (4.86 ± 1.07 versus 3.98 ± 0.73 mmol/L, *P* < 0.001), LDL-c (3.49 ± 0.98 versus 1.99 ± 0.62 mmol/L, *P* < 0.001), Lp(a) (288.04 ± 254.59 versus 187.94 ± 181.37 mmol/L, *P* = 0.004) and lower HDL-c (0.66 ± 0.24 versus 1.12 ± 0.3 mmol/L, *P* < 0.001). Apo A-1 was correlated to Lp(a) (*r* = 0.291, *P* = 0.005). Corticoid dose was not associated to dyslipidaemia, but in multiple regression models, corticoid dose may be negatively related to some atherogenic markers, in particular non-HDL-c. Tunisian patients with markedly active RA experience substantially reduced serum HDL-c and increased TC, LDL-c and Lp(a) concentrations as well as increased TC/HDL-c, LDL-c/HDL-c and non-HDL-c/HDL-c ratios.

RA	Lee et al. 2000 [22]	Nine (42.3%) of 21 RA patients and 6 (31.6%) of 19 controls had high Lp(a) levels (>30 mg/dL) and the Lp(a) level was higher in RA patients compared with controls (27.1 ± 5.3 versus 19.0 ± 4.2 mg/dL) without significant difference (*P* > 0.05).

RA	Park et al. 1999 [64]	Our study suggests that patients with untreated active RA have altered lipoprotein and apolipoprotein patterns that may possibly expose them to higher risk of atherosclerosis. The inflammatory condition of RA may affect the metabolism of HDL-cholesterol and apo A1.

RA	Rantapää-Dahlqvist et al. 1997 [65]	Lipoprotein(a), (Lp(a)), an independent atherogenic factor, was significantly increased in 93 patients with classical, seropositive rheumatoid arthritis of median disease activity. In the patients with Lp(a) concentrations above the upper reference value of 480 mg/L there was a significant correlation between Lp(a) and the concentration of orosomucoid, erythrocyte sedimentation rate, and the platelet count.

RA	Schultz et al. 2010 [66]	Inhibition of IL-6 signalling improves insulin sensitivity in humans with immunological disease suggesting that elevated IL-6 levels in type 2 diabetic subjects might be causally involved in the pathogenesis of insulin resistance. Furthermore, our data indicate that inhibition of IL-6 signalling decreases Lp(a) serum levels, which might reduce the cardiovascular risk of human subjects.

RA	Wang et al. 2008 [24]	Lp(a) levels were highest in active RA 259.01 ± 148.96 mg/dL) modest in controls (177.43 ± 106.51 mg/dL) and lowest in inactive RA (173.03 ± 106.20 mg/dL). Lp(a) concentrations were found positively correlated with ox-Lp(a) (*R* = 0.37, Pb 0.001) and Lp(a)-IC (*R* = 0.39, Pb 0.001) concentrations respectively. Ox-Lp(a) concentrations were also related with Lp(a)-IC concentrations (*R* = 0.64, Pb 0.001).

RA SLE	Zhang 2011 [26]	Serum beta(2)-GPI-Lp(a) (1.12 ± 0.25 U/mL versus 0.87 ± 0.19 U/mL, *P* < 0.0001) and beta(2)-GPI-ox-LDL (1.01 ± 0.20 U/mL versus 0.80 ± 0.08 U/mL, *P* < 0.0001) concentrations in RA were both significantly higher than those of controls. Ox-Lp(a) (8.38 ± 6.69 mg/L versus 5.49 ± 4.31 mg/L, *P* < 0.05) and ox-LDL (0.68 ± 0.65 mg/L versus 0.37 ± 0.13 mg/L, *P* = 0.001) were also higher in RA than in controls.

Ssc	Cerinic 2003 [18]	Coagulation was significantly activated in Ssc patients (increase in F1 + 2, *P* < .001; TAT, *P* < 0.01; and Lp(a), *P* < 0.05). Endothelial injury reduces endothelial function, as suggested by impairment of fibrinolysis and activation of the coagulative pathway.

SSc	Lippi et al. 2006 [20]	SSc patients had statistically significant differences when compared to healthy controls in median and 25–75th percentile distribution of Lp(a) (110 mg/L, 51–389 mg/L versus 79 mg/L, 29–149 mg/L; *P* = 0.005). When compared to current NCEP/AHA/ACC goals, the values distributions and the relative percentage of patients with undesirable or abnormal vales were statistically different for Lp(a) (29% versus 3%) and Hs-CRP (42% versus 12%) (both *P* < 0.001). Lp(a) measurement might be useful in SSc to identify and eventually treat subsets of patients more predisposed to develop thrombotic complications.

SLE autoimmune disease	Frostegård 2002 [2]	AD patients (SLE) are at high risk of CVD. Nontraditional risk factors like OxLDL, oxLDL autoantibodies, phospholipids, and inflammation could lead to new therapeutic strategies and insight into disease mechanisms.

SLE	Borba et al. 1994 [67]	Lp(a) levels are significantly higher in patients with SLE and are not influenced by disease activity, thrombosis, aCL, and steroid therapy.

SLE	George et al. 1999 [68]	Patients with SLE and venous thrombosis had higher levels of beta2GPI-IC when compared with thrombosis-free patients or with healthy controls (*P* < 0.05). Patients with higher Lp(a) levels (>50 mg/dL) possessed higher levels of beta2GPI-IC as compared with patients with lower Lp(a) concentration (<20 mg/dL) (*P* < 0.05).

SLE	Okawa-Takatsuji et al. 1996 [29]	Our study is the first to reveal that hypoalbuminemia appearing during disease flare plays an important role in increasing the serum Lp(a) levels in lupus patients with renal disease and shows that corticosteroid treatment reduced the elevated serum Lp(a) levels almost to original levels.

SLE	Romero et al. 1999 [13]	Existence of autoantibodies against MDA-Lp(a). The presence of antibodies reacting not only against MDA-LDL but also against MDA-Lp(a) supports the hypothesis of a role for oxidative phenomena in the pathogenesis of APS and atherosclerosis.

SLE autoimmune disease	Sari et al. 2002 [3]	Serum Lp(a) levels are significantly higher (*P* < 0.01) in patients with SLE, these patients have a risk of developing cardiovascular disease and atherosclerosis and should be followed up.

SLE	Zhang et al. 2010 [28]	Lp(a) (400 ± 213 mg/L versus 181 ± 70 mg/L) and ox-Lp(a) (27.07 ± 22.30 mg/L versus 8.20 ± 4.55 mg/L) concentrations were higher in SLE patients than in controls (*P* < 0.0001). Beta(2)-GPI-Lp(a) complexes were detectable in both controls and with higher levels in SLE patients.

Immune	Borberg 2006 [54]	Lp(a)-apheresis is available as a specific, highly efficient elimination procedure superior to techniques which also eliminate Lp(a).

Immune	Milioti et al. 2008 [4]	Antigenic stimuli in the pathogenesis of atherosclerosis: oxLDLs, beta2glycoprotein1 (beta2GP1), LP(a), heat shock proteins (HSPs), extracellular matrix components (collagen and fibrinogen), and foreign antigens including bacteria.

Autoimmune disease	Jonasson et al. 1997 [1]	Atherosclerosis, especially early onset coronary atherosclerosis, is not a disease associated with particular HLA alleles.
